# Controlled Cre/loxP Site-Specific Recombination in the Developing Brain in Medaka Fish, *Oryzias latipes*

**DOI:** 10.1371/journal.pone.0066597

**Published:** 2013-06-25

**Authors:** Teruhiro Okuyama, Yasuko Isoe, Masahito Hoki, Yuji Suehiro, Genki Yamagishi, Kiyoshi Naruse, Masato Kinoshita, Yasuhiro Kamei, Atushi Shimizu, Takeo Kubo, Hideaki Takeuchi

**Affiliations:** 1 Department of Biological Sciences, Graduate School of Science, The University of Tokyo, Tokyo, Japan; 2 National Institute for Basic Biology, Myodaiji, Okazaki, Aichi, Japan; 3 Department of Physiology, Tokyo Women's Medical University School of Medicine, Tokyo, Japan; 4 Division of Applied Biosciences, Graduate School of Agriculture, Kyoto University, Kyoto, Japan; 5 Department of Molecular Biology, Keio University School of Medicine, Tokyo, Japan; National University of Singapore, Singapore

## Abstract

**Background:**

Genetic mosaic techniques have been used to visualize and/or genetically modify a neuronal subpopulation within complex neural circuits in various animals. Neural populations available for mosaic analysis, however, are limited in the vertebrate brain.

**Methodology/Principal Findings:**

To establish methodology to genetically manipulate neural circuits in medaka, we first created two transgenic (Tg) medaka lines, Tg (*HSP*:*Cre*) and Tg (*HuC*:loxP*-DsRed-*loxP*-GFP*). We confirmed medaka *HuC* promoter-derived expression of the reporter gene in juvenile medaka whole brain, and in neuronal precursor cells in the adult brain. We then demonstrated that stochastic recombination can be induced by micro-injection of Cre mRNA into Tg (*HuC*:loxP*-DsRed-*loxP*-GFP*) embryos at the 1-cell stage, which allowed us to visualize some subpopulations of GFP-positive cells in compartmentalized regions of the telencephalon in the adult medaka brain. This finding suggested that the distribution of clonally-related cells derived from single or a few progenitor cells was restricted to a compartmentalized region. Heat treatment of Tg(*HSP*:*Cre* x *HuC*:loxP*-DsRed-*loxP*-GFP*) embryos (0–1 day post fertilization [dpf]) in a thermalcycler (39°C) led to Cre/loxP recombination in the whole brain. The recombination efficiency was notably low when using 2–3 dpf embyos compared with 0–1 dpf embryos, indicating the possibility of stage-dependent sensitivity of heat-inducible recombination. Finally, using an infrared laser-evoked gene operator (IR-LEGO) system, heat shock induced in a micro area in the developing brains led to visualization of clonally-related cells in both juvenile and adult medaka fish.

**Conclusions/Significance:**

We established a noninvasive method to control Cre/loxP site-specific recombination in the developing nervous system in medaka fish. This method will broaden the neural population available for mosaic analyses and allow for lineage tracing of the vertebrate nervous system in both juvenile and adult stages.

## Introduction

Genetic mosaic analysis is a powerful tool in the fields of neuroscience and developmental biology for labeling a subset of neurons, tracing cell-lineage, and modulating neuronal function [1]–[4]. The recent combination of Cre/loxP and optogenetic tools allows for specific modulation of selected neurons within complex neural tissues [5], [6]. *Cre* induction can be spatially controlled by cell type-specific promoters/enhancers and site-specific viral infection [7], [8]. The lack of appropriate promoters/enhancers, however, limits the neural population available for mosaic analysis in the vertebrate brain. Site-specific viral infection requires invasive surgical procedures, which also limits free access to the entire brain. Thus, the development of noninvasive methods for controlled Cre/loxP site-specific recombination will increase the neural population available for mosaic analyses of the vertebrate nervous system. To address this issue, we focused on a heat-inducible Cre/loxP gene induction system in medaka fish. Medaka embryos have high temperature tolerance (4–35°C) compared to zebrafish (25–33°C) [9], allowing for various temperature-mediated treatments [10]. In addition, an artificial heat-shock promoter (HSP) comprising multimerized heat shock elements has very low background activity and no leak in medaka fish [11], which allows for spatiotemporal site-specific *Cre* induction. Local heat treatment using a metal probe and an infrared laser results in ectopic *Cre* induction in a small number of cells in various tissues such as the gonads and epidermal tissues in medaka fish [12]–[14]. To our knowledge, however, there are no reports of its application in neural tissue.

Here we used an infrared laser-evoked gene operator (IR-LEGO) system to induce highly regulated spatiotemporal *Cre* expression in neural precursor cells of medaka embryos [15]. Medaka fish have a transparent chorion that facilitates noninvasive observations of the nervous system throughout development [10], and it allows for heating a small subpopulation of differentiating neurons in the neural placode using an infrared laser (wavelength: 1480 nm). Based on the fate maps of neural placodes in the medaka embryo, analysis of clonally related cells in a specific brain region of interest can be performed by irradiating a small population of the neuronal precursor cells.

To examine whether a heat-inducible Cre/loxP gene induction system works in the medaka nervous system, we generated transgenic (Tg) medaka lines for the detection of Cre/loxP recombination with the promoter regions of medaka *HuC*. *HuC* belongs to a family of vertebrate neuronal-specific genes homologous to the Drosophila *elav* and serves as an early marker of differentiating neurons [16], [17]. Zebrafish *HuC* is expressed in neuronal precursor cells during embryogenesis, and then high expression levels persist in most regions of post-hatching and larval brain [18], [19]. Thus, the zebrafish *HuC* promoter is widely used for visualizing and/or modifying the function of neural circuits in juvenile fish [20]. The *HuC* promoter is also applicable for visualizing the differentiation process during adult neurogenesis, as *HuC* expression is restricted to newborn and differentiating neurons in the adult zebrafish brain [19], [21]. In the present study, we demonstrated that stochastic Cre recombination during embryogenesis allowed for visualization of clonally-related cells in compartmentalized regions of the adult medaka brain. Further, irradiation of the developing medaka embryo brains using an infrared laser allowed for visualization of clonally-related HuC-expressing cells in both juvenile and adult medaka fish.

## Materials and Methods

### Ethics statement

The work in this paper was conducted using protocols approved by the Animal Care and Use Committee of the University of Tokyo (permit number: 12–07). All surgery was performed under cold anesthesia, and all efforts were made to minimize suffering.

### Fish and breeding conditions

Medaka fish (*Oryzias latipes*, drR strain) and all Tg lines, Tg (*HuC*:loxP-*DsRed*-loxP-*GFP)* and Tg (*HSP*:*Cre*), were maintained in their respective groups in plastic aquariums (12 cm ×13 cm ×19 cm). All fish were hatched and bred in our laboratory. The water temperature was maintained at ∼28°C and the light was provided by fluorescent lamps for 14 h per day (08∶00 to 22∶00).

### Generation of Tg (*HuC*:loxP-*DsRed*-loxP-*GFP*)

The medaka *HuC* promoter was estimated by comparing the medaka genome with the *HuC* promoter of zebrafish. The medaka *HuC* promoter fragments were polymerase chain reaction-amplified from bacterial artificial chromosome containing the medaka *HuC* genomic region (clone name; ola1-o12-D12, NBRP medaka) with KOD DNA polymerase (TOYOBO) and the following sets of primers carrying the indicated restriction enzyme sites (underlined): 5'-CCGCTCGAGCGGTTTTGTTGCACCGCTAATGTTAGG-3' and 5'-TCCCCGCGGGGAGTACAATGAAAGAAATCTAGGTCC-3', which contain the recognition sites for *Xho*I and *Sac*II, respectively. ploxP-*DsRed*-loxP-*EGFP* plasmid was obtained from Prof. Tanaka (National Institute for Basic Biology) [14].

### Generation of Tg (*HSP*:*Cre*/*Crs*:*BFP*)

The dual expression vector, pPBIS19-*mgfc*:Tag*BFP*-8x*HSE*:*Cre* containing fused gene of Cre recombinase with N-terminal nuclear localization sequence and the red fluorescent protein mCherry under the control of artificial heat shock inducible promoter [11]. This plasmid also had TagBFP (Wako Junyaku Kogyo Co. Ltd., Osaka, Japan) gene under the control of a mouse *gamma F crystalline* promoter [22] for selecting the proper transgenic lines. It allowed us to select the embryos carrying *HSP*:*Cre* transgene by blue fluorescence of their eyes without heat treatment. Insulators were inserted between the polyA sequence under the control of *HSP* promoter and TR3, and also inserted into the boundary region of the two promoters, *HSP* and *Crystalline* to prevent position-effects of the transgenes. The F1 embryos from wild type and vector-injected F0 were raised to sexual maturity and screened for germline transmission by a fluorescent microscopy examining BFP expression.

### Observation of fluorescent signals in brains slices of Tg line (*HuC*:loxP-*DsRed*-loxP-*GFP*)

Fixed brains were washed in phosphate buffered saline (PBS) containing 0.5% Triton (PBST) and incubated in Sca*/*eA2 solution (4 M urea, 10% (wt/vol) glycerol and 0.1% (wt/vol) Triton X-100 [23]) at room temperature for 3 h. The brains were washed again in 0.5% PBST and embedded in 4% agarose gel/PBS. Coronal slices (120 µm thick) were then cut with Vibroslicer (VT 1000 S, Leica). Fluorescent signals were imaged using a confocal laser-scanning microscope (LSM710; Zeiss). Volume-rendered images were displayed using FluoRender (http://www.fluorender.com).

### Immunohistochemistry

Immunostaining was performed on 14-µm cryosections. Whole brains were embedded in OCT compound (Sakura Tissue Tek) and cut using Cryostat (Leica, CM 1850). The sections were blocked with 0.2%Triton, 1% dimethylsulfoxide, and 2% bovine serum albumin in PBS at room temperature for 1 h, then incubated in the primary antibodies diluted in the blocking buffer at 4°C overnight. Primary antibodies used in this study were mouse anti-HuC/D (1∶500, Molecular Probes), rabbit anti-DsRed (1∶500, Clontech). Primary antibodies were detected by subclass-specific secondary antibodies labeled with Alexa 488/548 (1∶1000, Molecular Probes), respectively.

### mRNA microinjection and microscopy

Cre-SV40 was cloned into a pGEM-T easy vector (Promega, Madison, WI). Template preparation and *in vitro* synthesis of mRNAs were performed as described previously [24]. Confocal and fluorescence microscopy analyses were performed using a Zeiss confocal microscope (LSM710; Carl Zeiss, Oberkochen, Germany) and a Leica epifluorescence microscope (MZFLIII; Leica, Tokyo, Japan). The micrographs were processed with Photoshop software (Adobe, San Jose, CA) and the projection of a confocal stack was rendered using FluoRender (http://www.fluorender.com).

### Heat-induction in the whole body

Ten embryos were placed into a tube with 200 µl medaka hatching buffer [25] and heated at 39°C for the prescribed number of hours in a thermalcycler (TProfessional Basic; Biometra).

### Heat induction in the telencephalon

Embryos were mounted at stage 24, dorsal side up, in a drop of 2% methylcellulose (M-0387; Sigma Chemical Co., St. Louis, MO) and observed using the 20× custom made objective lens (mono-coated lens of UPlanSApo; Olympus, Tokyo, Japan) for an Olympus epifluorescence microscope with IR-LEGO unit (IR-LEGO 1000; Sigma-Koki, Saitama, Japan). Heat was induced in the telencephalon by 1480-nm light generated by a high-power single mode CW Raman fiber laser (Model PYL-3-1480-M; IRE-Polus Group, Sturbridge, MA), as previously described [12], [15].

## Results

### Generation and characterization of the *HuC*:loxP-*DsRed*-loxP-*GFP* Tg medaka line

**Figure 1:**
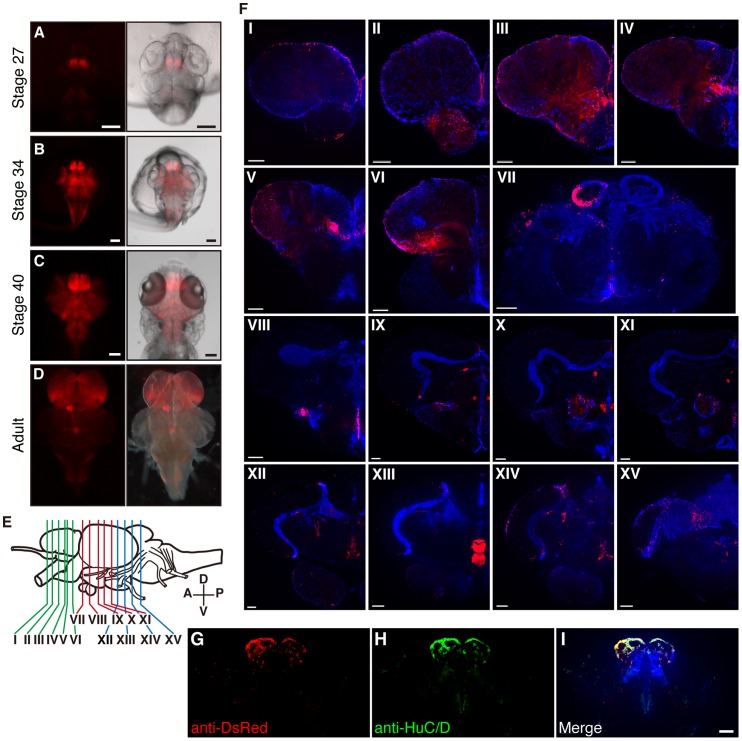
DsRed expression in *HuC*:loxP-*DsRed*-loxP-*GFP* Tg medaka at different stages. (A–D) Onset of DsRed expression in the brain from the dorsal view. DsRed expression was restricted to the anterior brain vesicle-intermediate brain vesicle (Ant-Int) at stage 27 (A). Additional DsRed-expressing neurons were observed in the entire brain at stages 34 (B) and 40 (C). At the adult stage (D), prominent DsRed expression was observed in the telencephalon and left side of the habenular nucleus. (E) Schematic drawing of adult medaka brain indicating the depth of each coronal section in (F). (F) Fluorescent images of coronal forebrain slices from an *HuC*:loxP-*DsRed*-loxP-*GFP* adult. Please see Fig. S1 for description of DeRed positive cells, by referring to the medaka brain atlas. (G–I) Photomicrographs depicting DsRed and HuC/D immunofluorescence within the habenular nucleus of the *HuC*:loxP-*DsRed*-loxP-*GFP* Tg brain. White arrow indicates a double-labeled neuron, which is shown separately below for DsRed (G) and HuC/D (H) immunofluorescence with DAPI (blue) staining, as well as in a composite (I). Scale bars 100 µm.

We generated a Tg medaka line that expresses loxP-*DsRed*-loxP-*GFP* under the control of a 3.3-kb medaka *HuC* promoter (*HuC*:loxP-*DsRed*-loxP-*GFP*). *DsRed* expression in the Tg embryo was first detected as early as stage 27 in the anterior brain vesicle-intermediate brain vesicle (Ant-Int; Fig. 1A and Fig. S1A). After stage 34, DsRed fluorescence was observed in the whole brain (Fig. 1B, C and Fig. S1B, C). In 3-month-old adult fish, DsRed-expressing cells were prominently detected in the superficial layer of the telencephalon, cerebellum, posterior edge of the optic tectum, and the left side of habenular nucleus (Fig. 1D, Fig. S2). To determine the details of *DsRed* expression, we prepared coronal sections of the Tg whole brain (*HuC*:loxP-*DsRed*-loxP-*GFP*; Fig. 1E, F). The surface of the telencephalon and the left side of habenular nucleus exhibited prominent DsRed expression (Fig. 1F panel I – VII). In addition, DsRed expression was detected in the olfactory bulb (panel II – IV), preoptic area (panel V), ventromedial nucleus (panel VII), optic tectum (panel VIII – XIV), marginal zone of the third ventricular zone (panel VIII – XI), hypothalamus (panel X – XII), and cerebellum (panel XIV – XV). We previously mapped “proliferation zones” comprising stem cells in the adult medaka brain [26] and found a large number of HuC-positive neural progenitors situated at or near the “proliferation zones” (Fig. S2). DsRed-positive axons were observed near the olfactory bulb, fasciculus longitudinalis medialis (Flm), nucleus glomerulosus posterioris (NGp), and corpus interpeduncularis (Ci), to which the habenular nucleus neurons project [27]. Taken together, these findings indicated that DsRed expression in the Tg (*HuC*:loxP-*DsRed*-loxP-*GFP*) labeled the neural progenitor cells with *huC* expression. Furthermore, to confirm that DsRed is expressed in HuC-positive neural progenitors, we performed co-staining with antibody against HuC/D and DsRed by immunohistochemistry. The pattern of HuC/D expression overlapped with that of DsRed, including the habenular nucleus (Fig. 1G–I).

### Stochastic Cre/loxP recombination by injection of a low concentration of Cre mRNA

**Figure 2:**
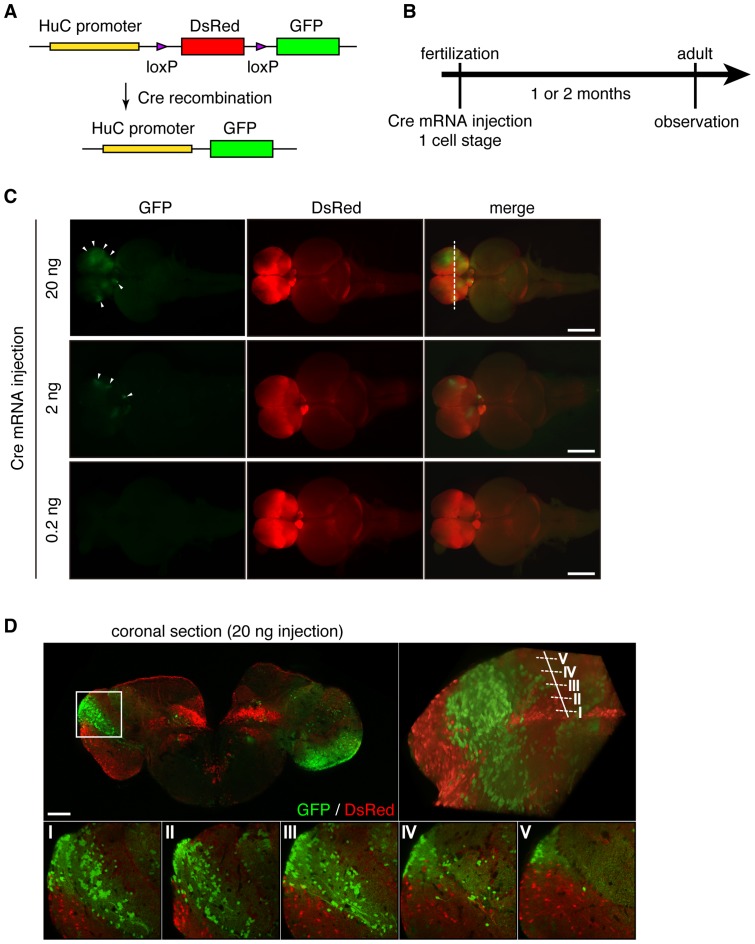
Cre mRNA-mediated gene recombination in the *HuC*:loxP-*DsRed*-loxP-*GFP* Tg brain. (A) *HuC*:loxP-*DsRed*-loxP-*GFP* construct. The gene recombination converts DsRed expression to GFP. (B) Experimental procedure. Cre mRNA was injected at the 1-cell stage and the fluorescence pattern was observed at the adult stage. (C) Fluorescent images of adult Tg embryos brain with Cre mRNA (20, 2, and 0.2 ng/µl, respectively) at the 1-cell stage. DsRed, enhanced GFP (EGFP), and merged images of embryos. Anterior is to the left, and right is toward the top. Scale bar, 1 mm. (D) Coronal fluorescent images of Cre mRNA (20 ng/µl)-injected brain. Merged images of DsRed and GFP in the posterior telencephalon. A magnified view of the lateral telencephalon (white box) shows the spatial compartmentalization of GFP-positive neurons in this region at 5 different depth levels (I–V). Scale bar, 100 µm.

We examined whether stochastic recombination can be induced by injection of Cre mRNA into the Tg embryos at the 1-cell stage, which would allow us to label the cell lineage. First, we confirmed that Cre recombination was induced in most cells of various tissues, including muscle and brain, by Cre mRNA injection (100 ng/µl) into Tg (*beta actin*:loxP-*DsRed*-loxP-*GFP*) under the control of a ubiquitous beta-actin promoter [7] (Fig. S3A-E). Next, a small amount of Cre mRNA (0.2, 2 and 20 ng/µl) was injected into the Tg (*HuC*:loxP-*DsRed*-loxP-*GFP*) at the 1-cell stage (30 min after fertilization). Recombination by Cre mRNA injection led to changes in the expression of DsRed to that of GFP (Fig. 2A). The mosaic expression of DsRed and GFP (white arrowhead) was detected at the adult stage (Fig. 2B, C). The induction of GFP expression increased dose-dependently. The distribution of GFP-expressing cells differed among individuals (Fig. S3F), indicating that stochastic recombination can be induced in some stem cells by the injection of Cre mRNA. We were unable to precisely determine the number of stem cells in which recombination occurred, however, because it takes at least 12 h to express mature GFP [28], during which multiple cell divisions may have occurred.

To visualize the three-dimensional distribution of the DsRed- and GFP-expressing cells, we obtained optical images of vibratome sections (120 µm) and generated three-dimensional images of the telencephalon of the Cre mRNA (20 ng/µl)-injected fish (Fig. 2C white dashed line, Fig. 2D, and Movie. S1). Interestingly, subpopulations of GFP-expressing cells were detected in spatially distinct segments. These findings suggest that subpopulations (defined as clonally-related cells) derive from a single or a few adjacent cells.

### Heat induction of Cre/loxP recombination in the nervous system

**Figure 3:**
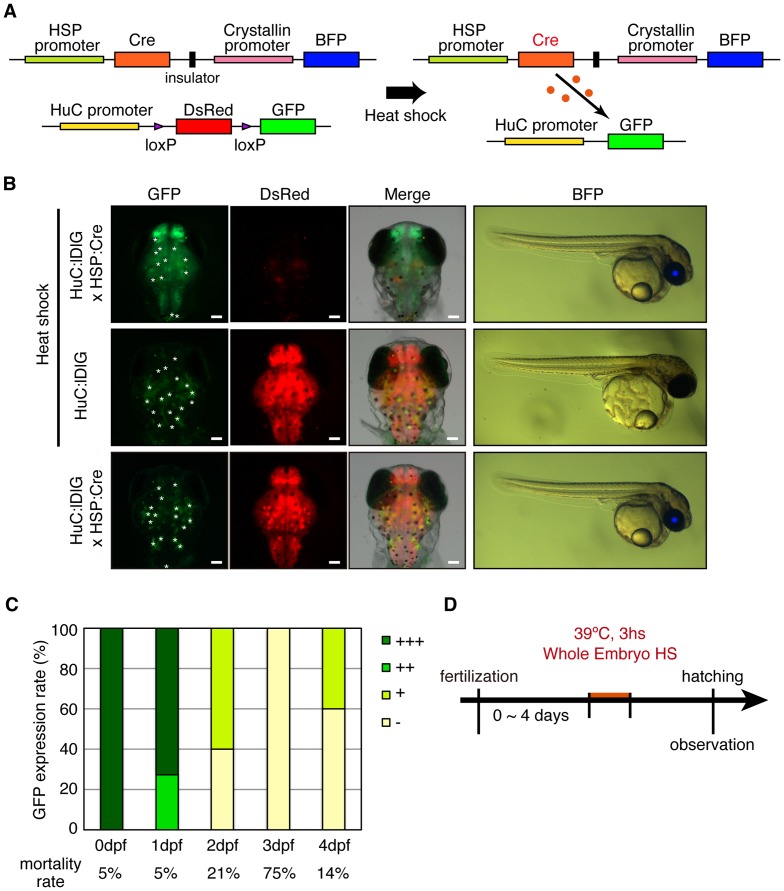
*Heat shock protein* (*HSP*) promoter-induced Cre/mediated recombination. (A) Schematic drawing of *HSP*:*Cry*/*Crs*:*BFP* constructs and mechanism of fluorescence change from DsRed to GFP in *HuC*:loxP-*DsRed*-loxP-*GFP* and *HSP*:*Cry*/*Crs*:*BFP* double Tg embryos. (B) Fluorescence color conversion by heat-induced Cre. *HuC*:loxP-*DsRed*-loxP-*GFP*/*HSP*:*Cre*/*Cry*:*BFP* (HuC:lDlG x HSP:Cre) embryos exposed to heat induction (39 C, 3 h) were observed under a stereoscopic fluorescence microscope just after hatching. DsRed expression was significantly reduced and GFP was induced (upper), compared to control embryos of the *HuC*:loxP-*DsRed*-loxP-*GFP* single Tg (HuC:lDlG, middle) and unheated double-Tg lines (lower). Blue eye (BFP) indicates the *HSP*:*Cre*/*Cry*:*BFP* transgene. Left columns are dorsal view images (top, anterior; right, right) and right columns are lateral view images (top, dorsal; right, anterior). Autofluorescence derived from pigment cells is indicated by asterisks in GFP panels. Scale bar, 100 µm. (C) The efficiency of heat-induced recombination and (D) Experimental procedure. The prominence of the fluorescence color conversion was categorized into four types (+++, strongest GFP fluorescence in the whole brain; ++, not so strong GFP fluorescence in the whole brain; +, GFP fluorescence in partial brain regions; -, no change).

To induce the recombination by heat treatment, we established a Cre-inducible Tg (*HSP*:*Cre*/*Cry*:*BFP*) with an artificial *HSP* promoter containing eight consecutive artificial heat-shock response elements and beta-globin minimum promoter derived from *Xenopus laevis*. Expression of Cre in the Tg (*HSP*:*Cre*) is reported to be inducible by a brief heat shock treatment. The construct expressed blue fluorescent protein (BFP) under the control of the mouse *Crystallin* (*Cry*) promoter as a screening marker, and an insulator sequence was inserted between *HSP*:*Cre* and *Cry*:*BFP* (Fig. 3A) [29] to avoid cross talk between the artificial *HSP* and *Cry* promoters which are located in the same construct of the vector. We generated Tg (*HuC*:loxP-*DsRed*-loxP-*GFP*/*HSP*:*Cre*/*Cry*:*BFP*) by genetic crossing.

We examined the efficiency of the heat-inducible recombination during embryonic development. We exposed the embryos to heat shock (39°C, 3 h) at the early blastula stage (stage 9–10, 0∼1 days post fertilization [dpf]). GFP fluorescence was detected at the time of hatching in the whole brain of Tg (*HuC*:loxP-*DsRed*-loxP-*GFP*/*HSP*:*Cre*/*Cry*:*BFP*) embryos (Fig. 3B, upper panel). In contrast, no GFP fluorescence was detected in either heat-exposed Tg (*HuC*:loxP-*DsRed*-loxP-*GFP*) (Fig. 3B, middle panel) or non-treated Tg (*HuC*:loxP-*DsRed*-loxP-*GFP*/*HSP*:*Cre*/*Cry*:*BFP*) embryos (Fig. 3B, lower panel). These findings indicated that Cre protein expressed by HSP in response to heat induction led to the recombination. In addition, the efficiency of the heat-induced recombination apparently decreased with development (Fig. 3C).

### Spacially controlled induction of Cre/loxP recombination using an IR-LEGO system

**Figure 4:**
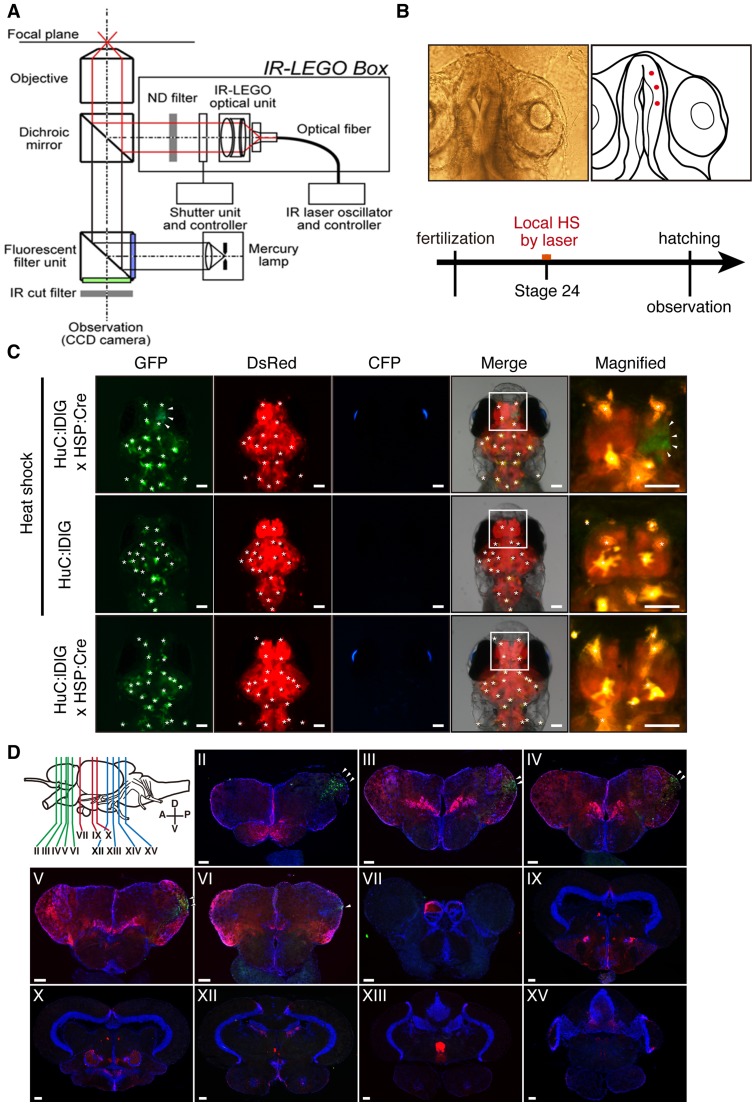
Spatial distribution of neural progenitor cell linage originating from a few neural stem cells in the telencephalon. (A) Schematic diagram of infrared laser-evoked gene operator (IR-LEGO) microscope system. An infrared laser beam (1480 nm) was introduced into the IR-LEGO optical unit through an optical fiber. Irradiation duration was controlled by a shutter unit. CCD, charge-coupled device; ND, neutral density. (B) Red dots indicate the point of infrared-laser irradiation (21.6 mW, 1 sec) at embryonic stage. Local heat shock by laser was performed at Stage 24 and the treated embryos were observed under a stereoscopic fluorescence microscope just after hatching (5 days after irradiation). (C) Fluorescence conversion from DsRed to GFP by IR-LEGO induced Cre in juvenile brain. The images show heat-treated *HuC*:loxP-*DsRed*-loxP-*GFP*/*HSP:Cre*/*Cry:BFP* (upper arrowheads), heat-treated *HuC*:loxP-*DsRed*-loxP-*GFP* (middle), and non-treated *HuC*:loxP-*DsRed*-loxP-*GFP*/*HSP:Cre*/*Cry:BFP* (lower). A magnified view of the telencephalon (white box) shows the spatial compartmentalization of GFP-positive neurons originating from Cre/induced neural stem cells. Yellow spots in which Green signals completely merged with DsRed signals represent autofluorescence of leucophores. Autofluorescence is indicated by asterisks in GFP panels. (D) Cross sectional analysis of GFP expression in the infrared-laser irradiated adult brain. GFP expression was seen only in right side. Scale bars, 100 µm.

To control Cre/loxP recombination spatially, we used the infrared laser-evoked gene operator (IR-LEGO) system (Fig. 4A) to induce local *Cre* expression by laser irradiation [12], [15]. We heated the surface of the right neural placode of the telencephalon in stage 24 Tg (*HuC*:loxP-*DsRed*-loxP-*GFP*/*HSP*:*Cre*/*Cry*:*BFP*) embryos (2 dpf, Fig. 1A) by irradiation with an incident laser power of 21.6 mW for 1 sec. GFP fluorescence was detected in a subpopulation of neurons in the right telencephalon in the heat-exposed Tg (*HuC*:loxP-*DsRed*-loxP-*GFP*/*HSP*:*Cre*/*Cry*:*BFP*) juvenile and adult fish only in the right side (Fig. 4C and 4D). The juveniles were observed after hatching. In negative controls, GFP fluorescence was not detected in either heat-exposed Tg (*HuC*:loxP-*DsRed*-loxP-*GFP*) or non-treated Tg (*HuC*:loxP-*DsRed*-loxP-*GFP*/*HSP*:*Cre*/*Cry*:*BFP*) fish (Fig. 4C) and also in the left side of the treated Tg. These findings indicated that GFP-expressing neurons in both juvenile and adult brains were clonally-related cells derived from the micro area of neural placodes exposed to the infrared laser.

## Discussion

The present study demonstrated that injection of a low concentration of Cre mRNA induces stochastic Cre/loxP recombination. There are two possible reasons for the stochastic Cre/loxP recombination. The first is that the injected mRNA may be sparsely distributed. The second is that a low concentration in the cells may cause stochastic recombination, which is consistent with previous reports showing that low transcriptional Cre activity leads to stochastic recombination [30]. In the present study, we were unable to demonstrate the recombination at a single cell level, perhaps because more time (>12 h) is required for cells to express detectable levels of GFP than the cell cycle time of neural progenitors [28]. Thus, we cannot exclude the possibility that GFP-expressing cells comprise more than one clonal unit derived from a single stem cell.

Next, we established a noninvasive method for controlled Cre/loxP site-specific recombination in the nervous system during medaka embryogenesis, which allowed us to visualize and/or modify the formation of a lineage-dependent structure. Recent studies with an advanced Gal4-UAS system in zebrafish promoted the genetic dissection of neural circuits [31], revealing how a subpopulation of neurons produces behaviors in juvenile fish [32]. The relationship between functional neural circuits and clonally-related neurons has, however, remained largely unknown. In the adult fruit fly brain, many areas of the brain neuropil are formed by the combination of distinct clonal units [33]–[35]. Recently, in the mouse visual cortex, clonally-related neurons derived from the same radial glia were found to have similar electrophysiologic properties (orientation selectivity) in response to a visual stimulus, in contrast to the response of the nearest-neighbor non-clonally related neurons [36], [37]. These findings suggest that clonally-related neurons, rather than identifiable macroscopic brain structures, constitute the functional modules of neural circuits. Several behavioral systems to assess optomotor response, schooling, shoaling, and mating partner preference were recently established in medaka fish [38]. Combinations of these behavioral systems and the present gene manipulation method will contribute to our understanding of how a subset of clonally-related neurons (a lineage-dependent structure) is involved in behavior.

**Figure 5:**
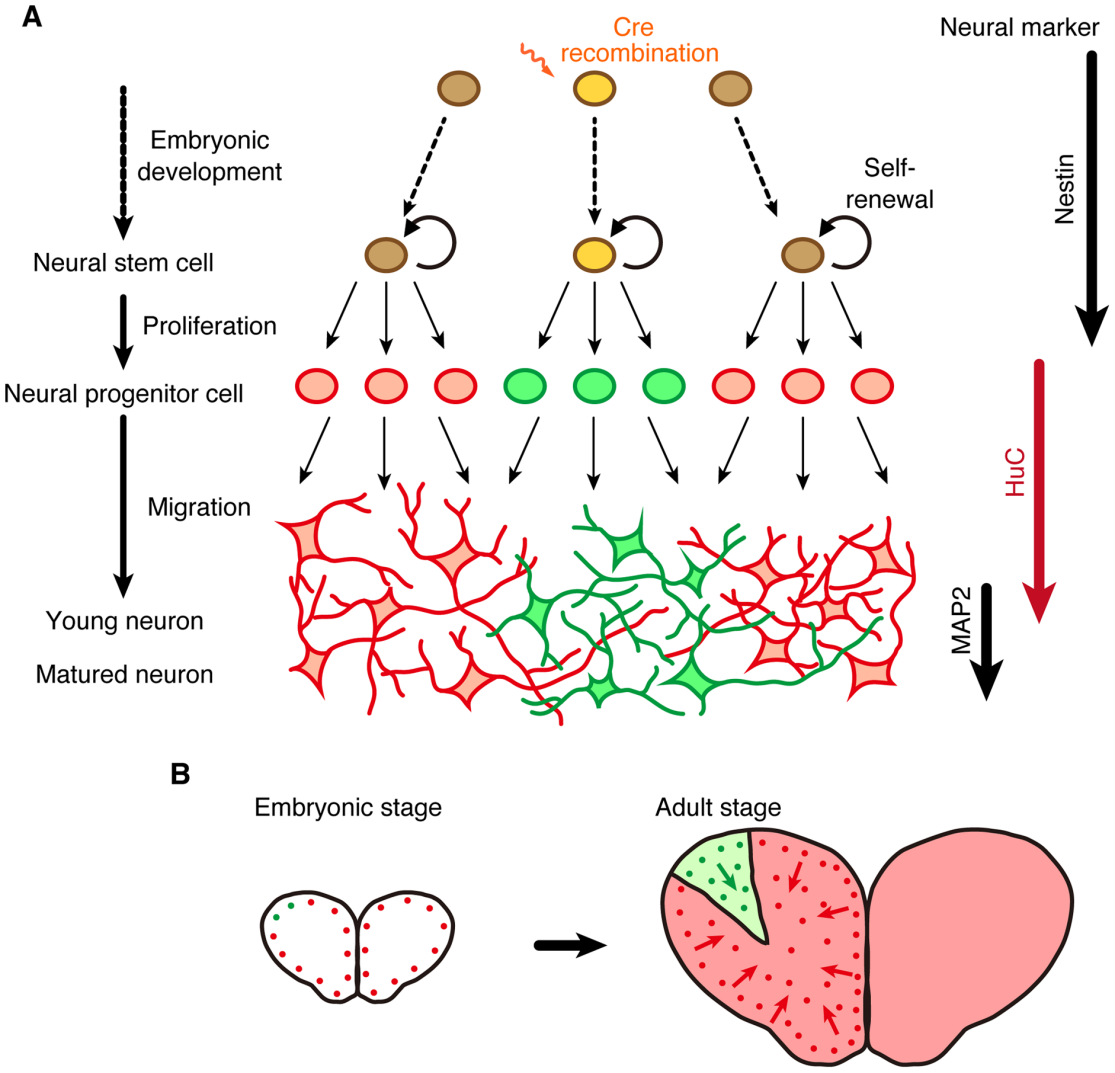
Compartmentalization of clonally-related neurons in the telencephalon. Site-specific Cre/loxP recombination in a single neural progenitor or small population of neural progenitors during embryonic development led to the formation of compartmentalized blocks comprising clonally-related neurons in the adult telencephalon.

Furthermore, in the adult brain, medaka *HuC* promoter activity is restricted to newborn and differentiating neurons. Thus, heat-inducible Cre/loxP site-specific recombination is also applicable for investigating adult neurogenesis in medaka fish. In contrast to mammals with limited neurogenesis in the adult brains, teleost fish such as medaka fish and zebrafish constitutively generate newborn neurons in numerous proliferating zones across the whole brain throughout life [21], [26], [39]–[41]. As the distribution of proliferation zones in the adult brain is mostly conserved among teleost fish, adult neurogenesis is believed to be important for the maintenance and development of the fundamental structure of the fish brain throughout life [21], [41]. Here we showed that stochastic and/or site-specific Cre/loxP recombination led to labeling of clonally-related neural progenitors that seem to form compartmentalized blocks in the telencephalon (Fig. 5). Although it is technically impossible to hit the same neuronal progenitor cells reproducibly, lineage analysis can be achieved by heat-induction in the same micro areas based on embryo morphology or the detection of fluorescent proteins. The use of a low laser intensity to theoretically target a single cell would allow us to induce recombination in a single or a few adjacent cells [15], [42]. Determination of the distribution of GFP-expressing cells by repeated analysis would enable us to identify clonal units derived from the target micro area. The presence of identical subpopulations of clonally-related cells in multiple samples could reveal clonal units. Considering that the GFP-expressing cells comprise a single, or possibly a few clonal units, all the distribution patterns could be described by a combination of identified clonal units. Thus, this work provides a promising method for lineage analysis of neural progenitors in the adult brain and allows for further investigation of the involvement of such lineage-dependent structures in the formation of functional neural circuits mediated by adult neurogenesis.

## Supporting Information

Figure S1 **DsRed expression in** ***HuC*** **: loxP-** ***DsRed*** **-loxP-** ***GFP*** **Tg medaka.** (A–C) Onset of DsRed expression in the brain from the lateral view at Stages 27 (A), 34 (B), and 40 (C). (D–G) Photomicrographs depicting DsRed (red), HuC/D (green), DAPI (blue), and their merged image immunofluorescence in the *HuC*: loxP-*DsRed*-loxP-*GFP* Tg brain in the preoptic area (D), optic tectum and hypothalamus (E, F), and cerebellum (G). Scale bar, 100 µm.
(TIFF)

Figure S2 **Schematic drawing of HuC-expressing neurons.** Magenta dots indicate the position of the HuC-expressing neurons. A, nucleus anterioris of diencephalon; AOT, tractus opticus (optic tract) axialis; Cb, corpus cerebelli; CbSg, stratum granulare of corpus cerebelli; CbSm, stratum moleculare of corpus cerebelli; CbSp, stratum Purkinje of corpus cerebelli; CM, corpus mamillare; D, area dorsalis telencephali; Dc, area centralis of D; Dl, area lateroposterioris of D; Dld, area laterodorsalis of D; Dlp, posterior subdivision of dorsolateral telencephalon; Dlv, area lateroventralis of D; Dm, area medialis of D; DOT, tractus opticus (optic tract) dorsalis; Dp, dorsal posterior telencephalon; DT, nucleus tegmentalis dorsalis; ECL, external cell layer of olfactory bulb; EP, epiphysis; ep, ependyme; Fll, fasciculus longitudinalis lateralis; Flm, fasciculus longitudinalis medialis; Flt, fasciculus longitudinalis lateralis telencephali; GL, glomerular layer of olfactory bulb; gc, griseum central; Hc, hypothalamus caudalis; Hd, nucleus dorsalis of habenula; HD, hypothalamus periventricularis dorsalis; Hv, nucleus ventralis of habenula; IQ, inferior oblique of nucleus of nervus oculomotorius; IR, inferior rectus of nucleus of nervus oculomotorius; MC, commissural minor; MOT, tractus opticus (optic tract) medialis; MR, medial rectus of nucleus of nervus oculomotorius; NCILP, nucleus centralis posterioris of lobus inferiosis; NDIL, nucleus diffusus of lobus inferioris; NDTL, nucleus diffusus of torus lateralis; NGp, nucleus glomerulosus medialis; ON, nervus olfactorius; OT, optic tectum; Pc, nucleus pretectalis centralis; PGc, nucleus preglomerulosus centralis; PGm, nucleus preglomerulosus medialis; PGZ, periventricular grey zone; PMp, nucleus preopticus magnocellularis pars parvocellularis; PPa, nucleus preopticus periventricularis, anterioris; PPp, nucleus preopticus parvocellularis posterioris; PSi, nucleus pretectalis superficialis pars intermedialis; PSm, nucleus pretectalis superficialis pars medialis; rp/V3, recessus preopticus of ventriculus tertius; RT, nucleus tegmentalis rostralis; SC, nucleus suprachiasmaticus; SR, superior rectus of nucleus of nervus oculomotorius; TA, nucleus tuberis posterioris; TCT, tractus cerebellotectalis; TIT, tractus isthmotectalis; TP, nucleus tuberis posterioris; TS, torus semicircularis; v3, ventriculus mesencephali; v4, ventriculus quartus; V, area ventralis of the telencephalon; VC, valvula cerebelli; Vd, area dorsalis of V; Vi, area intermedialis of V; Vl, ventral telencephalon, lateral subdivision; VL, nucleus ventrolateralis; VM, nucleus ventromedialis; vm, ventriculus mesencephali; VOT, tractus opticus (optic tract) ventralis; Vp, ventral telencephalon, posterior subdivision; Vv, area ventralis of V.
(TIFF)

Figure S3 **Cre/loxP recombination by Cre mRNA injection.** (A) Schematic drawing of Cre/loxP recombination in Tg (*beta actin*:loxP-*DsRed-*loxP-*GFP*). (B–F) Prominent GFP expression was observed in the whole body of Cre mRNA-injected embryos. Cre mRNA-injected embryos (B,D) and negative control embryos (C,E) are shown from dorsal (B,C) and lateral (D,E) views. Scale bar, 100 µm. (F) Mosaic pattern of GFP fluorescence in the Cre mRNA-injected Tg (*HuC*: loxP-*DsRed-*loxP-*GFP*) adult medaka brain. Different GFP patterns were observed in individual brains. Scale bar, 1 mm.
(TIFF)

Movie S1 **Serial optical sections corresponding to** **Fig 2D**.
(AVI)
