# Supplementary figures and images for: Controlled Cre/loxP Site-Specific Recombination in the Developing Brain in Medaka Fish, Oryzias latipes
Source: PLoS One. 2013 Jun 25;8(6):e66597. doi: 10.1371/journal.pone.0066597 (PMC3692484; doi:10.1371/journal.pone.0066597)

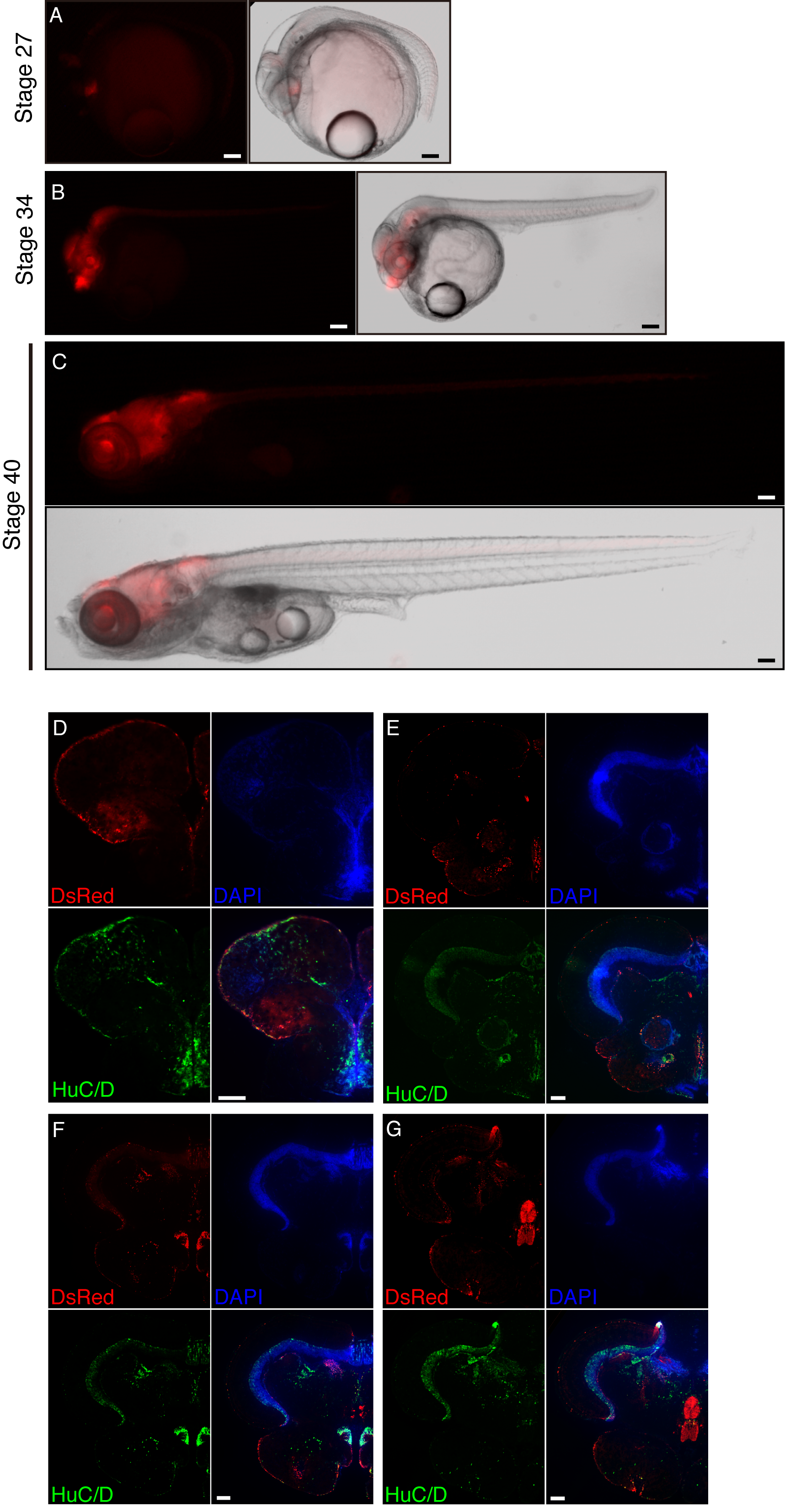

Supplement: Figure S1 — DsRed expression in HuC : loxP- DsRed -loxP- GFP Tg medaka. (A–C) Onset of DsRed expression in the brain from the lateral view at Stages 27 (A), 34 (B), and 40 (C). (D–G) Photomicrographs depicting DsRed (red), HuC/D (green), DAPI (blue), and their merged image immunofluorescence in the HuC: loxP-DsRed-loxP-GFP Tg brain in the preoptic area (D), optic tectum and hypothalamus (E, F), and cerebellum (G). Scale bar, 100 µm. (TIFF) [file pone.0066597.s001.tiff]

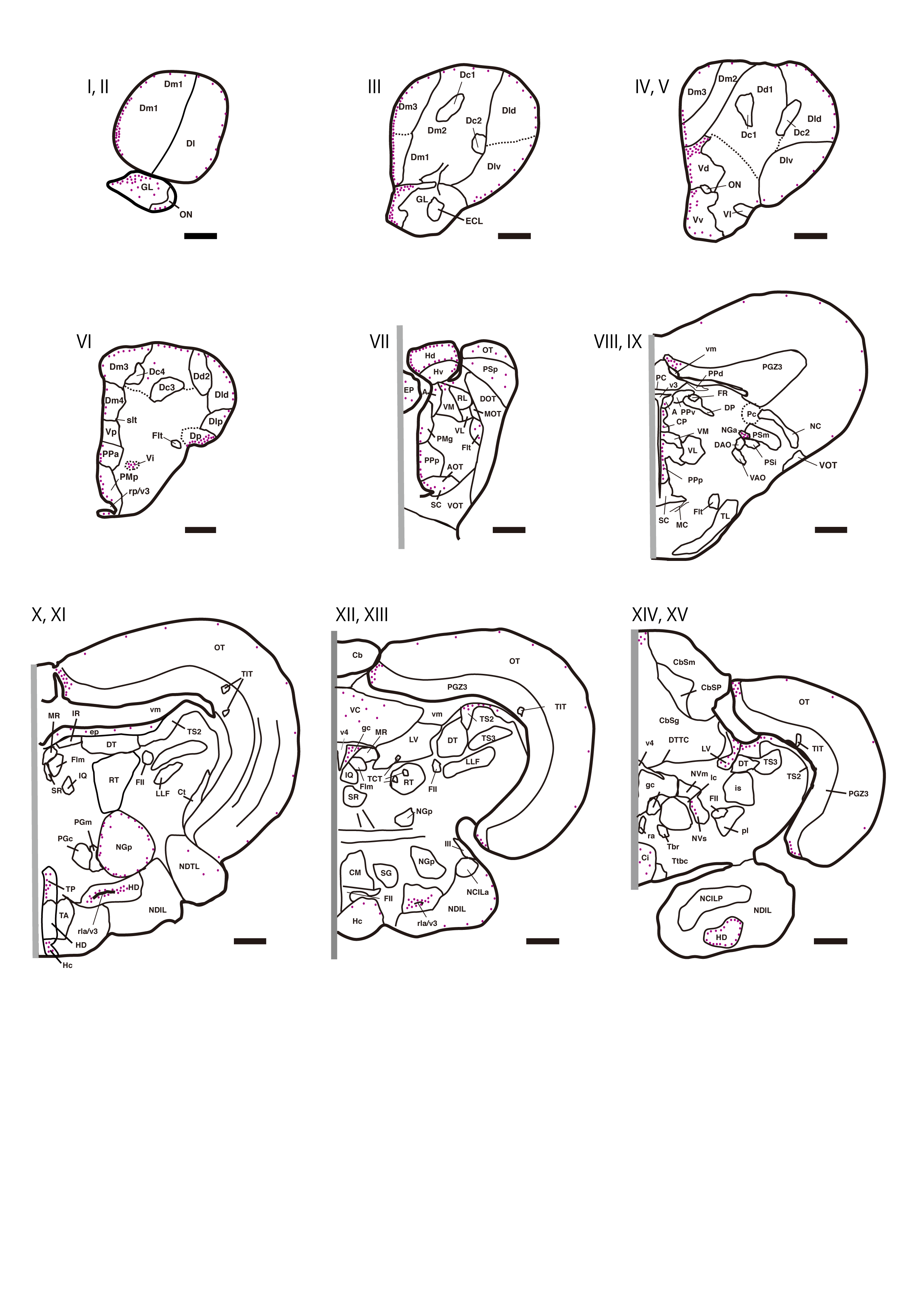

Supplement: Figure S2 — Schematic drawing of HuC-expressing neurons. Magenta dots indicate the position of the HuC-expressing neurons. A, nucleus anterioris of diencephalon; AOT, tractus opticus (optic tract) axialis; Cb, corpus cerebelli; CbSg, stratum granulare of corpus cerebelli; CbSm, stratum moleculare of corpus cerebelli; CbSp, stratum Purkinje of corpus cerebelli; CM, corpus mamillare; D, area dorsalis telencephali; Dc, area centralis of D; Dl, area lateroposterioris of D; Dld, area laterodorsalis of D; Dlp, posterior subdivision of dorsolateral telencephalon; Dlv, area lateroventralis of D; Dm, area medialis of D; DOT, tractus opticus (optic tract) dorsalis; Dp, dorsal posterior telencephalon; DT, nucleus tegmentalis dorsalis; ECL, external cell layer of olfactory bulb; EP, epiphysis; ep, ependyme; Fll, fasciculus longitudinalis lateralis; Flm, fasciculus longitudinalis medialis; Flt, fasciculus longitudinalis lateralis telencephali; GL, glomerular layer of olfactory bulb; gc, griseum central; Hc, hypothalamus caudalis; Hd, nucleus dorsalis of habenula; HD, hypothalamus periventricularis dorsalis; Hv, nucleus ventralis of habenula; IQ, inferior oblique of nucleus of nervus oculomotorius; IR, inferior rectus of nucleus of nervus oculomotorius; MC, commissural minor; MOT, tractus opticus (optic tract) medialis; MR, medial rectus of nucleus of nervus oculomotorius; NCILP, nucleus centralis posterioris of lobus inferiosis; NDIL, nucleus diffusus of lobus inferioris; NDTL, nucleus diffusus of torus lateralis; NGp, nucleus glomerulosus medialis; ON, nervus olfactorius; OT, optic tectum; Pc, nucleus pretectalis centralis; PGc, nucleus preglomerulosus centralis; PGm, nucleus preglomerulosus medialis; PGZ, periventricular grey zone; PMp, nucleus preopticus magnocellularis pars parvocellularis; PPa, nucleus preopticus periventricularis, anterioris; PPp, nucleus preopticus parvocellularis posterioris; PSi, nucleus pretectalis superficialis pars intermedialis; PSm, nucleus pretectalis superfi [file pone.0066597.s002.tiff]

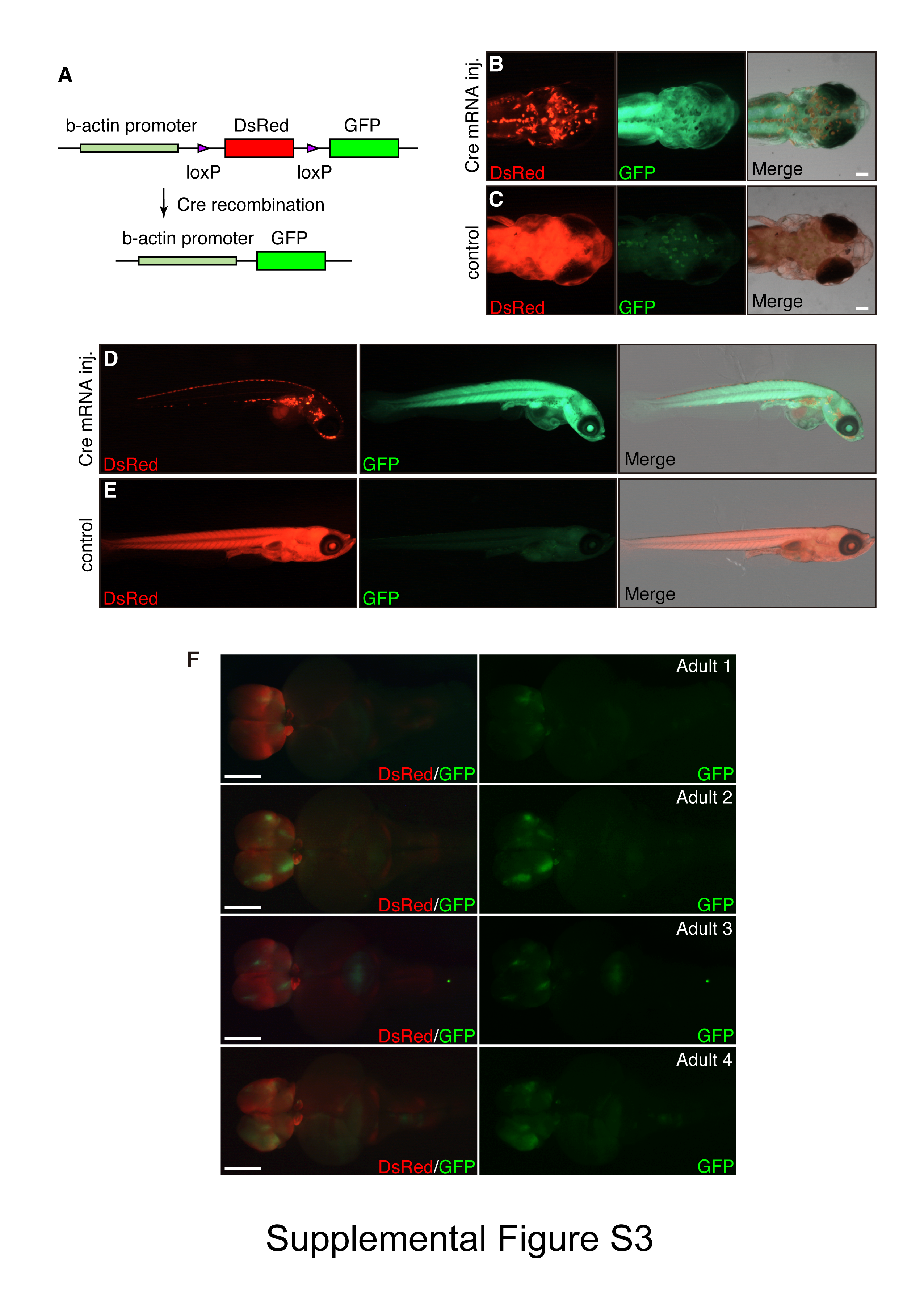

Supplement: Figure S3 — Cre/loxP recombination by Cre mRNA injection. (A) Schematic drawing of Cre/loxP recombination in Tg (beta actin:loxP-DsRed-loxP-GFP). (B–F) Prominent GFP expression was observed in the whole body of Cre mRNA-injected embryos. Cre mRNA-injected embryos (B,D) and negative control embryos (C,E) are shown from dorsal (B,C) and lateral (D,E) views. Scale bar, 100 µm. (F) Mosaic pattern of GFP fluorescence in the Cre mRNA-injected Tg (HuC: loxP-DsRed-loxP-GFP) adult medaka brain. Different GFP patterns were observed in individual brains. Scale bar, 1 mm. (TIFF) [file pone.0066597.s003.tiff]
